# Stocking density effects on turkey hen performance to 11 weeks of age

**DOI:** 10.1016/j.psj.2022.101874

**Published:** 2022-03-24

**Authors:** S. Jhetam, K. Buchynski, T. Shynkaruk, K. Schwean-Lardner

**Affiliations:** Department of Animal and Poultry Science, University of Saskatchewan, Saskatoon, SK, Canada, S7N 5A8

**Keywords:** body weight, feed efficiency, mortality, uniformity, air quality

## Abstract

Stocking density (**SD**) affects economic return for turkey production and can impact performance parameters. In this study (2 experimental blocks), Nicholas Select hens (n = 3,550 poults/block) were randomly placed in 1 of 4 SD treatments of 30, 40, 50, or 60 kg/m^2^ in open rooms (67.5 m^2^) with 4 replications per treatment. Feeder and drinker space were equalized on a per bird basis. Air quality was measured, and ventilation was adjusted to equalize ammonia and carbon dioxide levels across all rooms. Group BW and feed consumption were measured on d 0 and wk 3, 5, 8, and 11. BW gain and mortality corrected feed-to-gain ratio were calculated. Mortality and culls were recorded daily and necropsied for cause of death. At wk 8 and 11, flock uniformity was evaluated (30 birds/replicate). Data were analyzed using regression analyses in SAS 9.4 (Proc Reg for linear regression and Proc RSReg for quadratic regression; SD as independent variable). An ANOVA was performed for air quality (Proc Mixed; SAS 9.4) and a Tukey's range test was used to separate means. Differences were considered significant when *P* ≤ 0.05. Carbon dioxide and ammonia were consistent across treatments for both blocks. At wk 11, BW decreased linearly as SD increased (*P* = 0.05). There was a tendency for overall BW gain to decrease linearly as SD increased (*P* = 0.06). Feed consumption decreased as SD increased during wk 8 to 11 (linear; *P* < 0.01) and from wk 0 to 11 (quadratic; *P* = 0.04). SD had no impact on feed efficiency, mortality, or uniformity. Total aggression related mortality and culls were highest in the 30 kg/m^2^ treatment (linear; *P* = 0.02). A brief economic analysis was performed utilizing commercial poult and feed costs and income at marketing. Net room income increased as SD increased (linear; *P* < 0.01). The results indicate that high SD negatively impacted turkey hen final BW and feed consumption, but no effect was observed on feed-to-gain ratio, percent mortality, or uniformity.

## INTRODUCTION

Stocking density (**SD**) has many effects on turkey production parameters, including performance, health, welfare, and producer profitability. It can have adverse effects on turkey performance such as BW, feed efficiency, and mortality. These effects can negatively impact economic return but can also indicate birds are experiencing stress or poor wellbeing. The majority of studies assessing SD in turkeys have focused on turkey toms. The most current literature on the effects of SD in turkey hens was published 22 yr ago. Though these studies provided valuable information, there have been many advances in genetic selection for improved growth rate and feed efficiency since then. When evaluating SD, some studies have altered group size and maintained floor space (Moran, 1985; Noll et al., 1991; Martrenchar et al., 1999; Beaulac and Schwean-Lardner, 2018; Beaulac et al., 2019) while others have maintained group size but altered pen size/floor space (Coleman and Leighton, 1969; Denbow et al., 1984; Leighton et al., 1985; Buchwalder and Huber-Eicher, 2004; Bartz et al., 2020). For the practical application of SD guidelines, altering group size rather than changing rearing facilities is more easily implemented on farm. The gaps in the literature for assessing SD effects on the performance and health of turkey hens demonstrates the importance of SD guidelines that are based on more current literature.

Though there has been more extensive research with broiler chickens, the available literature focusing on turkeys found that as SD increased, BW of toms and hens decreased (Coleman and Leighton, 1969; Proudfoot et al., 1979; Leighton et al., 1985; Moran, 1985; Noll et al., 1991; Martrenchar et al., 1999; Beaulac et al., 2019; Bartz et al., 2020). However, the effects on feed efficiency varied between studies. Some studies found that increasing SD resulted in poorer feed efficiency (Leighton et al., 1985; Noll et al., 1991; Beaulac et al., 2019; Bartz et al., 2020) and others found no effect on feed efficiency (Coleman and Leighton, 1969; Proudfoot et al., 1979). Stocking density has shown few negative effects on mortality of turkeys in previous literature, with only numerical differences or tendencies (*P* < 0.06) for higher mortality rates with increasing SD observed in the studies by Coleman and Leighton (1969) and Noll et al. (1991) respectively. However, numerical differences are important to note as statistically significant results may not accurately depict the effects of SD on bird mortality, as low mortality rates and variability between treatment rooms or other confounding factors make determination of significance difficult when discussing mortality (Beaulac et al., 2019).

Poorer performance in birds reared at higher densities may be a result of increased stress due to changes in group size, reduced floor space and mobility, competition at the feeder, or changes in air quality and litter moisture (Beaulac et al., 2019). Litter and air quality are affected by higher SD as more birds produce larger quantities of fecal matter, which contribute to wet litter and poor air quality. Ammonia is released as a result of microbial decomposition of poultry manure (Ritz et al., 2004) and carbon dioxide (**CO_2_**) levels can also be directly related to SD due to increased respiratory output (Zuidhof et al., 1993). Ammonia levels exceeding 10 ppm affect the health of poultry (Nagaraja et al., 1983; Schwean-Lardner et al., 2013) and are associated with respirable dust particles (Wathes et al., 1997; Ritz et al., 2004). The effects of litter and air quality on birds are important confounding factors to consider when evaluating the impacts of SD.

The objectives of this research were to evaluate the impact of graded levels of SD on turkey hen performance to 11 wk of age to assist in improving SD guidelines for commercial turkey production. This study also aimed to eliminate certain confounding factors by controlling air quality and equalizing feeder and drinker space between all treatments. Finally, this study provides a basic economic analysis of the graded levels of SD evaluated. It was hypothesized that high SD levels would negatively affect BW, feed efficiency, mortality, and flock uniformity due to increased stress and reduced space allowance. It was also hypothesized that economic returns would increase with increasing SD.

## MATERIALS AND METHODS

The experimental procedures for this experiment were approved by the University of Saskatchewan Animal Care Committee and all birds were cared for as specified in the Guide to the Care and Use of Farm Animals in Research, Teaching, and Testing by the (Canadian Council on Animal Care, 2009). This study was part of a larger research study that evaluated the impacts of SD on turkey hen performance, health, and welfare. The primary objective of this manuscript focused on the effects of SD on turkey hen performance and environmental quality, however, the data regarding the effects of SD on turkey hen behavior, health, and welfare have also been reported (Jhetam et al., 2020, 2021; Jhetam, 2021).

### Experimental Design

Four SD treatments (30, 40, 50, and 60 kg/m^2^) were arranged in a randomized complete block design, with 2 trial blocks allowing for increased replication. Each trial (block) consisted of 2 room replicates per SD treatment. The study was conducted at the University of Saskatchewan Poultry Centre in a floor housing facility that includes separate, and independently controlled rooms for environmental parameters.

### Birds and Housing

Nicholas Select turkey hens were obtained from a commercial hatchery and were infrared beak and toe treated. Poults (n = 3,550/block) were randomly selected and placed in 1 of the 4 SD treatments. The number of birds placed in each treatment was calculated according to the final predicted BW of 7 kg for turkey hens at 11 wk of age (Aviagen, 2015a). An additional 5% of birds were placed per room to account for predicted mortality, in an effort to ensure the final target SD were reached at 11 wk of age. The number of poults placed was 295, 388, 482, and 571 per room for the final predicted SD treatments of 30, 40, 50, and 60 kg/m^2^, respectively.

Birds were housed in large open rooms (6.7 × 10.0 m = 67.5 m^2^). Brooder rings (7.0 m in diameter) with wood shaving bedding (7–10 cm thick) and heat lamps were used for the first 10 d. Wheat straw (10–13 cm depth) was utilized for bedding during the rearing period. Feed was provided ad libitum using aluminum tube feeders with a pan diameter of 36 cm for the first 40 d and a large pan diameter of 44 cm for the remaining time. During the first 7 d, humidifiers were utilized to maintain relative humidity at a minimum of 50% (Aviagen, 2015b). Water was provided through Lubing EasyLine pendulum turkey nipple drinkers (Lubing, Cleveland, TN). Feeder and drinker space were equalized on a per bird basis for each SD treatment (35 birds/feeder; 30 birds/nipple) to eliminate impacts of feeder and drinker space. Birds were fed a commercial 5-phase diet (Table 1) in specific quantities per bird which included a starter 1 (1.4 kg/bird), starter 2 (1.8 kg/bird), grower 1 (2.8 kg/bird), grower 2 (3.8 kg/bird), and a finisher diet (2.2 kg/bird). Supplemental feeders and drinkers were provided throughout the first 10 d. Diet changes were made when the pre-determined amount of each ration was finished, and the total feed amount was adjusted at each diet change to account for mortality. Environmental enrichment was supplied throughout the trials by providing intact straw bales (1 bale/90 birds). Bales were replaced when they were destroyed and the straw was spread throughout the room, thus, no additional litter management was applied.

**Table 1:** Nutrient content per kilogram of diet fed to turkey hens from 0 to 11 wk of age.

Nutrient	Starter 1	Starter 2	Grower 1	Grower 2	Finisher
ME^1^ (kcal/kg)	3015	3089	3142	3229	3276
Crude protein (%)	28.2	26.0	25.0	21.9	20.5
Crude fat (%)	7.4	7.6	7.9	8.1	8.2
Crude fiber (%)	3.3	3.2	3.3	3.0	3.0
Chloride (%)	0.3	0.3	0.2	0.2	0.2
Calcium (%)	1.5	1.4	1.2	1.2	1.0
Phosphorus-total (%)	1.0	1.0	0.9	0.8	0.8
Sodium chloride (%)	0.3	0.3	0.2	0.2	0.2
Sodium (%)	0.2	0.2	0.2	0.2	0.2
Lysine-DP^2^ (%)	1.7	1.5	1.4	1.2	1.1
Methionine-DP (%)	1.2	0.7	0.6	0.5	0.5
Methionine + Cystine-DP (%)	1.5	1.0	0.9	0.8	0.7
Threonine-DP (%)	1.0	0.9	0.8	0.7	0.7
Selenium (mg/kg)	0.3	0.3	0.3	0.3	0.3
Vitamin A (KIU/kg)	12.0	12.0	12.0	10.0	10.0
Vitamin D_3_ (KIU/kg)	4.4	4.4	4.4	4.5	4.5
Vitamin E (IU/kg)	100.0	100.0	100.0	60.0	60.0

1 ME, metabolizable energy.

2 DP, digestible protein.

**Figure 1:**
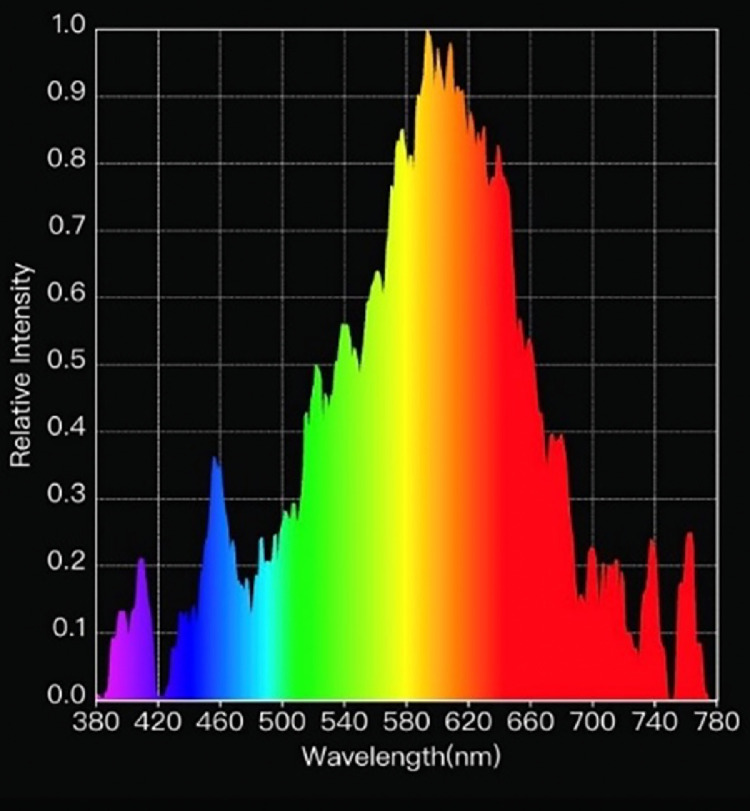
Wavelength composition (nm) of white light provided in the study.

During wk 1, room temperature was set at 28°C. Heat lamps were also provided over the brooder rings for the first 10 d. Temperature decreased by approximately 1°C each wk to a target temperature of 16°C by wk 11. Daylength and light intensity were 23L:1D and 40 lux, respectively, on d 1 and were gradually reduced to a final daylength and light intensity of 18L:6D and 5 lux, respectively, by d 9. Light was provided by LED (light emitting diode) white (1,100 lumens; Figure 1) dimmable bulbs (11W ALIS Non-Directional LED Lamps, Greengage Agritech Ltd, Roslin Innovation Centre, Midlothian, UK). A 15-min dawn and dusk period was applied throughout the trial by gradually increasing or decreasing light intensity prior to lights turning on or off.

Mortality and morbidity were monitored daily, and birds were culled when necessary due to illness and/or skeletal or growth abnormalities. From d 1 to 3, all mortalities and culls were replaced with spare poults in an attempt to maintain the final predicted SD. In block 1, additional space was blocked off at wk 3 to account for high mortality (caused by unanticipated yolk sac infections in the poults during wk 1) during the first 3 wk of the trial. At wk 8, mortality rates were low, and space was opened to maintain the estimated final SD for each treatment. In block 2, birds were removed from each treatment at wk 9 due to low mortality up to that age, to achieve the final predicted SD at wk 11.

### Data Collection

Body weight and feed consumption were measured by collecting group (room basis) BW and feeder weights on d 0 and wk 3, 5, 8, and 11. Feed consumption and mortality corrected feed-to-gain ratio (**F:G^m^**) were calculated for each of these time periods. Flock uniformity was determined by individually weighing a subsample of birds (30 birds/replication) at wk 8 and 11. After the bird replacement period (d 1–3), all mortality and culls were recorded daily and sent for necropsy to an independent diagnostic laboratory, and all mortality and morbidity results were then categorized by cause (Table 2). Basic economic analyses were performed to determine net income from both blocks by utilizing commercial poult cost, commercial feed costs, and income from bird sales to a commercial processing company and including variables such as number of birds placed, average final BW, and number of birds marketed for each SD treatment.

**Table 2:** Mortality and culls diagnosis categories.

Category	Diagnosis
Aggression	Head/neck pecked, wing pecked, and/or snood pulled
Metabolic	Ascites, chronic heart failure, right ventricular heart disease, round heart disease, slipped tendon, aortic rupture, peri-renal hemorrhage, hemorrhagic fatty liver syndrome
Infectious	Arthritis, synovitis, cellulitis, hepatitis, endocarditis, pericarditis, peritonitis, splenitis, keel bursitis, bursitis, enlarged hock joints
Unknown	No visible lesions
Mechanical	Broken wing, broken leg, ruptured tendon, trauma
Skeletal	Rickets, valgus varus, rotated tibia, spondylolisthesis, tibial dyschondroplasia
Other	Impaction, hepatomegaly, lateral tibial tarsal ligament rupture, enlarged kidney, enlarged spleen

Room temperatures were monitored hourly for the duration of both blocks using iButton Hygrochron temperature and humidity data loggers (Maxim Integrated; San Jose, CA) and average weekly room temperatures were calculated over the course of each block. From d 1, carbon dioxide (CO_2_) was measured 3 times per wk using a handheld CO_2_ meter (CO_2_40; Extech Instruments; Nashua, NH) and ammonia was monitored once per wk until differences were noted, then twice per wk. Ammonia was measured using Dräger-Tubes and a handheld pump (Draeger, Inc.; Houston, TX). If CO_2_ levels varied by 20% or ammonia differed by 5 ppm between rooms, ventilation was adjusted in each individual room to match air quality between all density treatments (Beaulac and Schwean-Lardner, 2018; Beaulac et al., 2019).

### Statistical Analyses

The experiment followed a randomized complete block design (trial as block) with rooms as the replicate unit. Data were checked for normality (Univariate Procedure) and all mortality data were log transformed (log +1) prior to regression analyses, and reported means were back transformed. Regression analyses were conducted using the Regression Procedure (Proc Reg) and Surface Response Regression Procedure (Proc RSReg) to determine if there were either linear or quadratic relationships between SD and the performance parameters being evaluated (SAS 9.4, Cary, NC). An ANOVA was performed for air quality and room temperature data using the Proc Mixed Procedure and Tukey's range test was used to separate means (SAS 9.4). Differences were considered significant if *P* ≤ 0.05 and trends were noted if *P* ≤ 0.10.

## RESULTS

All results are presented in terms of the final estimated SD. The actual SD achieved at 3, 5, 8, and 11 wk of age is shown in Table 3. At 11 wk of age, the average final SD achieved was 31.70, 42.38, 52.01, and 61.33 kg/m^2^.

**Table 3:** Actual stocking densities (kg/m^2^) achieved at 3, 5, 8, and 11 wk of age.

Age (wk)	n	Stocking density (kg/m^2^)			
		30	40	50	60
3	4	3.44	4.57	5.65	6.65
5	4	8.87	12.07	14.95	17.59
8	4	21.25	28.66	35.13	41.51
11	4	31.70	42.38	52.01	61.33

### Body Weight

At placement, poult BW was similar across all treatments and no differences in BW were observed at wk 3, 5, or 8 (Table 4). At 11 wk of age, turkey hen BW demonstrated a linear decrease (*P* = 0.05) as SD increased (8.36, 8.35, 8.30, 8.19 kg for SD treatments 30, 40, 50, and 60 kg/m^2^, respectively). Stocking density did not affect turkey hen BW gain for 0–3, 3–5, 5–8, and 8–11 wk (Table 4). Overall BW gain from 0 to 11 wk of age demonstrated a linear tendency (*P* = 0.06) to decrease with increasing SD.

**Table 4:** Effect of estimated final stocking density on turkey hen body weight and body weight gain (kg) at 0, 3, 5, 8, and 11 wk of age.

Age (wk)	n	Stocking density (kg/m^2^)				SEM^1^	*P*-value (Linear)	*P*-value (Quadratic)	Regression equation^2^
		30	40	50	60				
Body weight									
0	4	0.058	0.059	0.058	0.058	0.001	0.93	0.88	-
3	4	0.80	0.80	0.79	0.79	0.003	0.19	0.88	-
5	4	2.09	2.13	2.12	2.10	0.015	0.87	0.87	-
8	4	5.06	5.12	5.05	5.03	0.030	0.54	0.50	-
11	4	8.36	8.35	8.30	8.19	0.033	0.05	0.44	Y = −0.56e^−2^x+8.55
Body weight gain									
0–3	4	0.74	0.74	0.73	0.73	0.002	0.12	0.89	-
3–5	4	1.29	1.34	1.33	1.31	0.013	0.64	0.25	-
5–8	4	2.97	2.99	2.92	2.93	0.038	0.60	0.89	-
8–11	4	3.31	3.22	3.26	3.16	0.029	0.14	0.93	-
0–11	4	8.31	8.29	8.25	8.13	0.033	0.06	0.44	-

1 Standard error of the mean.

2 Regression considered significant if *P* ≤ 0.05.

### Feed Consumption and Feed Efficiency

Turkey hen feed consumption from 0 to 3, 3 to 5, and 5 to 8 wk of age was not affected by SD (Table 5). From 8 to 11 wk of age, feed consumption linearly decreased (*P* < 0.01) as SD increased (7.50, 7.47, 7.38, 7.21 kg for SD treatments 30, 40, 50, and 60 kg/m^2^, respectively). Overall feed consumption, from 0 to 11 wk of age, demonstrated a quadratic relationship (*P* = 0.04) with the lowest feed consumption in the 60 kg/m^2^. Mortality corrected F:G ratio was unaffected by SD for 0–3, 3–5, 5–8, 8–11, and 0–11 wk of age (Table 5).

**Table 5:** Effect of estimated final stocking density on turkey hen feed consumption (kg per bird) and mortality corrected feed-to-gain ratio from 0 to 3, 3 to 5, 5 to 8, 8 to 11, and 0 to 11 wk of age.

Age (wk)	n	Stocking density (kg/m^2^)				SEM^1^	*P*-value (Linear)	*P*-value (Quadratic)	Regression equation^2^
		30	40	50	60				
Feed consumption									
0–3	4	0.88	0.88	0.88	0.88	0.004	0.69	0.99	-
3–5	4	1.85	1.90	1.87	1.86	0.029	0.74	0.22	-
5–8	4	4.97	5.04	4.95	4.95	0.050	0.79	0.75	-
8–11	4	7.50	7.47	7.38	7.21	0.041	<0.01	0.32	Y = −0.94e^−2^x+7.81
0–11	4	15.19	15.29	15.08	14.90	0.055	0.01	0.04	Y = −0.88e-^3^ x2 +0.067x+14.00
Feed-to-gain mortality corrected (F:G^m^)									
0–3	4	1.18	1.19	1.19	1.19	0.006	0.72	0.93	-
3–5	4	1.43	1.42	1.41	1.42	0.016	0.23	0.24	-
5–8	4	1.67	1.68	1.69	1.69	0.005	0.20	0.64	-
8–11	4	2.68	2.74	2.68	2.72	0.110	0.94	0.96	-
0–11	4	1.90	1.92	1.90	1.91	0.024	0.95	0.87	-

1 Standard error of the mean.

2 Regression considered significant if *P* ≤ 0.05.

### Mortality

Total mortality and culls as a percentage of turkey hens placed was not affected by SD (Table 6). Total mortality and culls categorized by cause showed significant results for the “other” category (foreign body, hepatomegaly, lateral tibial tarsal ligament rupture, enlarged kidney, enlarged spleen) and for aggression (Table 7). Total mortality and culls in the “other” category were highest (linear; *P* = 0.03) in the low SD treatment of 30 kg/m^2^ (1.02, 0.97, 0.57, 0.66% for SD treatments 30, 40, 50, and 60 kg/m^2^, respectively). Total aggression related mortality and culls were highest in the low SD of 30 kg/m^2^ (linear; *P* = 0.02) and lowest in the 60 kg/m^2^ treatment (0.68, 0.58, 0.52, 0.09% for SD treatments 30, 40, 50, and 60 kg/m^2^, respectively).

**Table 6:** Effect of estimated final stocking density on turkey hen percent mortality and culls (%) from 1 to 3 wk, 3 to 5, 5 to 8, 8 to 11, and 0 to 11 wk of age.

Age (wk)	n	Stocking density (kg/m^2^)				SEM^1^	*P*-value (Linear)	*P*-value (Quadratic)	Regression equation^2^
		30	40	50	60				
1–3^3^	4	4.32	3.86	4.72	5.39	1.087	0.94	0.79	-
3–5	4	1.53	1.42	1.14	0.83	0.278	0.56	0.63	-
5–8	4	0.76	0.90	0.83	1.05	0.106	0.26	0.95	-
8–11	4	1.44	1.29	1.92	1.23	0.217	0.88	0.55	-
0–11	4	8.05	7.47	8.61	8.49	1.592	0.86	0.93	-

1 Standard error of the mean.

2 Regression considered significant if *P* ≤ 0.05.

3 Wk 1 started on d 3 when poults were no longer being replaced to maintain final targeted stocking density.

**Table 7:** Effect of estimated final stocking density on turkey hen percent mortality and culls (% of birds placed) by cause from day 3 to 11 wk of age.

Cause^3^	n	Stocking density (kg/m^2^)				SEM^1^	*P*-value (Linear)	*P*-value (Quadratic)	Regression equation^2^
		30	40	50	60				
Metabolic	4	0.34	0.39	0.78	0.70	0.096	0.08	0.85	-
Skeletal	4	0.59	0.39	0.52	0.53	0.118	0.84	0.70	-
Infectious	4	4.66	3.99	5.08	5.56	1.213	0.80	0.87	-
Unknown	4	0.59	1.03	1.09	0.96	0.198	0.61	0.37	-
Other	4	1.02	0.97	0.57	0.66	0.075	0.03	0.57	Y = −0.015x+1.47
Mechanical	4	0.08	0.13	0.10	0	0.043	0.48	0.44	-
Aggression	4	0.68	0.58	0.52	0.09	0.103	0.02	0.28	Y = −0.018x+1.29

1 Standard error of the mean.

2 Regression considered significant if *P* ≤ 0.05.

3 **Metabolic**: ascites, chronic heart failure, right ventricular heart disease, round heart disease, slipped tendon, aortic rupture, peri-renal hemorrhage, hemorrhagic fatty liver syndrome; **Skeletal**: rickets, valgus varus, rotated tibia, kinky back, tibial dyschondroplasia; **Infectious:** yolk sac infection, arthritis, synovitis, cellulitis, hepatitis, endocarditis, pericarditis, peritonitis, splenitis, bursitis, enlarged hock joints; **Unknown**: no visible lesion; **Other**: foreign body, hepatomegaly, lateral tibial tarsal ligament rupture, enlarged kidney, enlarged spleen; **Mechanical**: broken wing, broken leg, ruptured tendon, trauma; **Aggression**: head/neck pecked, wing pecked, snood pulled.

### Flock Uniformity

Turkey hen flock uniformity, presented as the percentage of birds found within 5, 10, or 15% of the mean room BW was not affected in relation to SD at 8 or 11 wk of age. At 8 wk of age, there were 43.33, 49.17, 47.50, and 48.33% of hens within 5%, 79.17, 75.83, 75.00, and 79.17% within 10%, and 90.83, 94.16, 93.33, and 88.33 within 15% of the mean for SD treatments 30, 40, 50, and 60 kg/m^2^, respectively. At 11 wk of age, there were 47.50, 43.33, 51.67, and 45.00% within 5%, 77.50, 80.83, 85.83, and 79.17% within 10%, and 93.33, 95.83, 93.33, 93.33% within 15% of the mean for SD treatments 30, 40, 50, and 60 kg/m^2^, respectively.

### Air Quality

Carbon dioxide and ammonia concentrations (ppm) for block 1 and block 2 are shown in Table 8. The average CO_2_ concentrations did not differ between treatments for either block (*P* = 0.70 and 0.24 for blocks 1 and 2, respectively). Similarly, average ammonia concentrations did not differ between treatments for both blocks (*P* = 0.06 and 0.32 for blocks 1 and 2, respectively). It is important to note that rapid increases in CO_2_ and ammonia occurred when external ambient temperatures were extremely low as both blocks took place during winter in Saskatchewan, Canada. This resulted in rapid increases in CO_2_ and ammonia as ventilation was reduced to maintain internal temperatures and prevent unwanted chilling of the birds. However, the rapid increases were accounted for by increasing the ventilation once the weather allowed. No differences were noted for average room temperature for each SD treatment from 1 to 11 wk (Table 9).

**Table 8:** Average room carbon dioxide (CO_2_) and ammonia concentrations (ppm) in relation to estimated stocking density over 11 wk.

Parameter (ppm)	N	Stocking density (kg/m^2^)				SEM^1^	*P*-value (ANOVA)^2^
		30	40	50	60		
Block 1							
Average CO_2_	2	1,976	1,990	2,030	2,010	18.50	0.70
CO_2_ range	2	326-4,058	356-3,820	475–4,211	463-3,907	-	-
Average ammonia	2	4.8	6.5	6.0	6.6	0.315	0.11
Ammonia range	2	0–15	0–12	0–25	0–25	-	-
Block 2							
Average CO_2_	2	1,958	1,998	2,059	2,076	26.12	0.24
CO_2_ range	2	463–4,561	634–4,537	584–4,089	661–3,997	-	-
Average ammonia	2	6.8	7.5	6.4	6.7	0.211	0.32
Ammonia range	2	0-30	0-25	0-25	0-24	-	-

1 Standard error of the mean.

2 ANOVA considered significant if *P* ≤ 0.05.

**Table 9:** Average weekly room temperature (°C) across estimated final stocking density treatments from 1 to 11 wk.

Age(wk)	n	Stocking density (kg/m^2^)				SEM^1^	*P*-value^2^ (ANOVA)
		30	40	50	60		
1	4	28.2	28.3	28.3	28.3	0.081	0.98
2	4	27.4	27.4	27.2	27.3	0.079	0.90
3	4	26.0	26.0	25.7	25.8	0.087	0.69
4	4	24.1	23.9	23.9	23.9	0.058	0.65
5	4	22.4	22.3	22.1	22.2	0.072	0.63
6	4	20.9	20.8	20.7	20.7	0.113	0.96
7	4	19.9	19.5	19.6	19.6	0.089	0.44
8	4	19.1	19.1	18.8	19.0	0.058	0.36
9	4	18.6	18.4	18.4	18.5	0.065	0.88
10	4	17.7	17.6	17.3	17.3	0.115	0.63
11	4	16.4	16.5	16.4	16.5	0.084	0.96

1 Standard error of the mean.

2 ANOVA considered significant if *P* ≤ 0.05.

### Economic Analysis

The basic economic analysis was performed using poult cost, feed cost, number of birds shipped, and bird meat income as seen in Table 10. The highest SD resulted in higher profits (linear; *P* < 0.01). The net income was CA$1706.88, CA$2283.41, CA$2723.20, CA$3144.22 per room replicate for the 30, 40, 50, and 60 kg/m^2^ treatments, respectively.

**Table 10:** Economic analyses of estimated final stocking density of turkey hens to 11 wk of age.

Parameter per room	n	Stocking density (kg/m^2^)				SEM^1^	*P*-value (Linear)	*P*-value (Quadratic)	Regression equation^2^
		30	40	50	60				
Number placed	4	295	388	482	571	-	-	-	-
Poult cost (CA$)^3^	4	604.75	795.40	988.10	1,170.55	-	-	-	-
Number shipped	4	248	330	404	479	-	-	-	-
Avg. final BW (kg)	4	8.36	8.35	8.30	8.19	0.033	0.05	0.44	Y = 0.0056x+8.55
Live wt. shipped (kg)	4	2,076.33	2,754.83	3,351.45	3,921.20	-	-	-	-
Bird meat income^4^	4	3,955.40	5,247.94	6,384.51	7,469.89	-	-	-	-
Feed intake (kg)	4	3,989.54	5,357.84	6,447.61	7,535.53	-	-	-	-
Feed cost (CA$)^5^	4	1,643.77	2,169.13	2,673.21	3,155.12	-	-	-	-
Net income per bird (CA$)	4	6.87	6.90	6.74	6.56	0.109	0.28	0.63	-
Net income per room (CA$)^6^	4	1,706.88	2,283.41	2,723.20	3,144.22	146.264	<0.01	0.48	Y = 47.52x+326.11

1 Standard error of the mean.

2 Regression considered significant if *P* ≤ 0.05.

3 Poult cost was CA$2.05/poult.

4 Meat price per kilogram live weight used was CA$1.905.

5 Feed price per tonne for Block 1 (Jan-Apr 2019): starter 1- CA$588; starter 2- CA$539; grower 1- CA$521; grower 2- CA$485; finisher- CA$460; Feed price per tonne for Block 2 (Nov 2019-Feb 2020): starter 1- CA$552; starter 2- CA$521; grower 1- CA$498; grower 2- CA$466; finisher- CA$440.

6 Net income = (Number shipped*Avg. BW* meat price) - (Number placed×Poult cost) - (Feed cost).

## DISCUSSION

In commercial poultry production, there are many factors that can influence performance parameters and economic return, and SD is one of those influencing factors. Body weight, feed efficiency, and mortality of the poultry being raised can directly affect economic return and thus, the effect SD has on those production traits are important to evaluate. With few studies evaluating the effects of SD in turkey hens, it is important to establish guidelines based on current research that allow for efficient production while balancing bird wellbeing. Currently, there are many variations in the SD guidelines for turkeys in North America. For example, in the United States the National Turkey Federation (2012) suggests a maximum SD of up 73.2 kg/m^2^, whereas the Certified Humane program suggests a maximum standard of 36.6 kg/m^2^ (Humane Farm Animal Care, 2014). Depending on the final predicted body weight of the turkeys raised, the Codes of Practice recommended a SD range from 40 to 65 kg/m^2^ (National Farm Animal Care Council, 2016). The Canadian Codes of Practice recommend a SD range of 45 kg/m^2^ up to 50 kg/m^2^ if specific environmental and management requirements are met for turkeys with a final BW of 6.2 to 10.8 kg (National Farm Animal Care Council, 2016), such as the turkey hens in this study. The differences found in SD recommendations may be a result of varying effects of SD on turkeys observed in previous literature. Therefore, it is important to develop guidelines based on current research, especially for turkey hens, as more studies have focused on toms.

Increasing SD resulted in a lower BW in turkey hens at 11 wk of age in this study, with a tendency for overall BW gain to reduce with increasing density. This effect on BW at older ages in hens and toms has been observed in previous studies (Coleman and Leighton, 1969; Proudfoot et al., 1979; Leighton et al., 1985; Noll et al., 1991; Martrenchar et al., 1997; Beaulac et al., 2019; Bartz et al., 2020). In these studies, as well as the current study, the number of feeders and drinkers were equalized on a per bird basis, which eliminates the effect of reduced feeder space which in itself could affect growth. In the study by Beaulac et al. (2019), the authors observed a decrease in BW and BW gain with increasing SD (30–60 kg/m^2^) at 12 and 16 wk of age when the final predicted SD was closer to being achieved. Similarly, the BW results from this study demonstrate differences at 11 wk of age when the target SD was achieved suggesting that older birds (11 wk and older) are more impacted by high SD resulting in slower growth. These effects on growth may be related to poor mobility, reduced floor space, and increased stress. Gait scores indicated that more birds in the 60 kg/m^2^ treatment had an identifiable abnormality that did not affect overall function (11 wk) and a higher incidence of footpad dermatitis (**FPD**; 8 and 11 wk) (Jhetam et al., 2020). The poorer mobility, presence of FPD, and increased litter moisture observed at 11 wk of age (Jhetam et al., 2020) could make reaching the feeder more difficult, especially because floor space becomes limited as the final estimated SD is more closely achieved. The birds’ wellbeing is affected by the presence of FPD as it has been associated with poor gait and may cause discomfort and pain (Martland, 1984; Weber Wyneken et al., 2015). Additionally, indicators of stress (heterophil to lymphocyte ratio) were higher for turkeys in the 50 and 60 kg/m^2^ treatments (Jhetam, 2021; Jhetam et al., 2021) at wk 11, and this may have negatively impacted BW. Broilers exposed to multiple stressors had reduced BW and poor feed efficiency from the reallocation of resources in the body from growth toward the stress response (McFarlane and Curtis, 1989; McFarlane et al., 1989).

The studies that have examined the effect of SD on feed consumption in turkeys have focused on turkey toms, however, it is an important parameter to examine as it directly affects economic return and growth. Previous studies have found that feed consumption in toms decreased as SD increased between various periods within the range of 12 to 20 wk of age (Leighton et al., 1985; Noll et al., 1991; Beaulac et al., 2019) and overall feed consumption from 0 to 20 wk (Noll et al., 1991). In accordance with previous literature, the results of this study found a linear decrease in feed consumption from 8 to 11 and overall feed consumption from 0 to 11 wk of age. Similar to the effects of SD on BW at high SD, feed consumption was not influenced by feeder or drinker space as it was equalized on a per bird basis which reduced the impact of feeder space on feed consumption. Feed consumption may have decreased due to poorer bird mobility, FPD (Jhetam et al., 2020), and difficulty reaching the feeders at older ages.

The difficulty of reaching the feeder is further supported by changes in behavior. Initially, at 8 wk of age, a larger percentage of birds in the 60 kg/m^2^ treatment were at the feeder despite a higher incidence of FPD (Jhetam, 2021; Jhetam et al., 2021). This suggests that birds in higher densities were motivated by social feeding behavior (Beaulac et al., 2019; Collins and Sumpter, 2007) and were still able to move to feeders when floor space was not as limited at that age. By 11 wk of age, when the final estimated SD was reached and floor space was limited, the percentage of birds present at the feeder did not differ between treatments (Jhetam, 2021; Jhetam et al., 2021), however, feed consumption decreased with increasing SD. Birds housed at high SD also rested more and fewer birds were observed walking suggesting that they may have been less motivated to access feeders as they would need to exert more energy to move between more resting pen mates (Beaulac and Schwean-Lardner, 2018; Beaulac et al., 2019).

Feed efficiency demonstrated no relationship with SD. Previous literature found that feed efficiency was poorer at high SD (60 kg/m^2^) from 4 wk of age and older in toms (Beaulac et al., 2019) which may have been related to increased stress and poorer feather cover and cleanliness of the toms in that treatment (Beaulac and Schwean-Lardner, 2018). Feed efficiency was also poorer at 8 wk of age, as well as in older hens and toms (40 and 61 kg/m^2^) (Leighton et al., 1985; Noll et al., 1991). These studies equalized feeder and drinker space on a per bird basis, thus, access to feeder space was not a confounding factor. Moran (1985) did not provide feeders and drinkers on a per bird basis and found feed efficiency to be negatively impacted by the highest SD in their study (21.4 kg/m^2^), indicating lack of feeder space as a confounding factor. However, there may have been other confounding factors contributing to the results observed in previous studies which would serve as stressors to the birds and may affect feed efficiency. In the current study, internal room temperature and air quality were consistent across treatments and ventilation was adjusted when differences were noted, which may help reduce the impact of SD on F:G^m^. Coleman and Leighton (1969) found poorer feed efficiency at high SD (48 kg/m^2^) in one of 2 of their experiments. The authors suggest that the effect seen in the first experiment at high SD was because it was conducted in winter with curtain sided barns compared to summer for the second experiment. Therefore, the amount of energy and feed required to thermoregulate and maintain body temperature would have resulted in poorer feed efficiency. Without confounding factors of room temperature and feeder space in this study, feed efficiency was less likely to be impacted by SD.

Overall mortality was unaffected by SD and this result is similar to previous literature. Coleman and Leighton (1969) saw no effect on mortality with increasing density (27.5–48 kg/m^2^), however, Noll et al. (1991) observed a tendency (*P* < 0.06) for higher mortality with increasing density (29.4–60.9 kg/m^2^). Beaulac et al. (2019) observed increased aggression related mortality and culls from wk 4 to 8 in toms and the authors suggest this may have been due to increased activity or increased frustration with large group size in the low and high SD treatments, respectively. Overall aggression related mortality and culls for the duration of this study increased with decreasing density. This may be related to behavioral changes observed at 8 wk of age such as increased walking, standing, litter pecking, and most evidentiary, the increase in aggressive behavior at low SD (Jhetam, 2021; Jhetam et al., 2021). Beaulac et al. (2019) observed a similar trend (*P* = 0.09) where aggression related mortality increased linearly with decreasing SD at wk 12 to 16 in toms. The authors suggest that birds at low SD may be more active compared to birds at high SD when floor space is reduced (Beaulac and Schwean-Lardner, 2018; Beaulac et al., 2019). To the best of the author's knowledge, the current study is the first to show an increase in aggression related mortality and culls at low SD in hens. This provides valuable information regarding the wellbeing of turkey hens housed at low SD and the social stressors affecting them.

When evaluating turkey hen percent mortality and culls by cause, the cause category of “other”, which includes death from a foreign body, hepatomegaly, lateral tibial tarsal ligament rupture, enlarged kidney, and enlarged spleen, demonstrated a linear increase with decreasing density. Statistical analysis did not show incidence of one condition within the ‘other’ category to be higher than another as these mortality causes occur at a low incidence. However, it may be important to understand why these conditions could occur when related to SD. Ligament ruptures in turkeys can be caused from trauma after physical activity, when birds move from a sitting to standing position, or stress on the hock joints and tendons from extreme weight (Crespo et al., 2002). During wk 5 to 8, metabolic related mortality and culls (such as ascites, heart disease, slipped tendon, and aortic rupture) were highest at high SD. Slipped tendons can cause lameness or present as bowed legs in turkeys with fast growing strains being more genetically susceptible to this condition which can result from trauma (Balloun, 1958; Julian, 1984). This may relate to increased disturbances observed in birds at high SD at 8 wk of age (Jhetam, 2021; Jhetam et al., 2021) when floor space starts to decrease, and birds would have to walk over a resting pen mate when maneuvering through the room during a significant growth period.

To the best of the author's knowledge, only one study has evaluated flock uniformity in relation to SD in turkeys (Beaulac et al., 2019). Flock uniformity was not impacted by SD in the current study, which is in accordance with the study by Beaulac et al. (2019). This may differ from broiler data. Broilers exhibited poorer uniformity at low SD but higher BW which may indicate that they grew to their genetic potential, whereas broilers at high SD were more uniform due to reduced space (Feddes et al., 2002). This may be because of social feeding behavior where birds are more likely to eat when others are present at the feeder which will result in more coordinated feeding at high SD (Collins and Sumpter, 2007; Beaulac et al., 2019). As no differences were observed in this study or the study in toms, this may suggest species differences or too few birds sampled (Beaulac et al., 2019).

Although economic return is greatest with higher SD, a balance between income (poult and feed cost vs. income only), production, health, and welfare parameters are important when developing SD guidelines to ensure the welfare of birds is maximized without compromising economic return. Additionally, this economic analysis did not consider management and labor costs, equipment damage, or potential carcass condemnations, thus not accurately depicting the true effects of high SD on economic return.

In conclusion, high SD negatively impacts some aspects of turkey hen performance to 11 wk of age. It was hypothesized that high SD would negatively affect BW, feed consumption, feed efficiency, and flock uniformity due to reduced space allowance and environmental stressors. It was also hypothesized that at low SD there would be more aggression. Although feed efficiency and uniformity were unaffected by SD, growth and feed consumption were negatively impacted at high SD and higher aggression related mortality and culls occurred at low SD. By assessing performance, health, and behavior, it is evident that a moderate density of around 40 to 50 kg/m^2^ is more beneficial to turkey hens raised to 11 wk of age as it ensures efficient production, health, and wellbeing. However, continuous monitoring of CO_2_ and ammonia is important to ensure that air quality or environmental conditions are not negatively affecting the health of the birds. This recommendation is based on maintaining good air quality by managing ventilation and barn temperatures.
